# Celastrol promotes DNA damage and apoptosis in uterine corpus endometrial carcinoma via promotion of KAT2B-mediated RBPJ acetylation and repression of MCM4 transcription

**DOI:** 10.1186/s10020-025-01082-z

**Published:** 2025-02-03

**Authors:** Lipeng Pei, Yan Zhu, Wenpeng Li, Bin Xu, Jingli Sun

**Affiliations:** 1 Department of Obstetrics and Gynecology, General Hospital of Northern Theater Command, No. 83, Wenhua Road, Shenhe District, Shenyang, 110016 China; 2 Department of Cardiovascular Surgery, General Hospital of Northern Theater Command, No. 83, Wenhua Road, Shenhe District, Shenyang, 110016 China

**Keywords:** Uterine corpus endometrial carcinoma, Celastrol, KAT2B, Acetylation, DNA damage, Apoptosis

## Abstract

**Background:**

Uterine corpus endometrial carcinoma (UCEC) is one of the most frequent female genital malignant tumors. Targeting DNA damage and cell apoptosis are regarded as effective ways for UCEC therapy. Celastrol is a natural anti-cancer product from the Celastraceae plant family, while its role in UCEC has not been investigated.

**Methods:**

UCEC cell lines Ishikawa and HEC-1-A were applied and treated with different concentrations of Celastrol. The appropriate and nontoxic concentrations were used for the subsequent experiments. Functional experiments analyzed the cell viability, cell cycle distribution, DNA damage, apoptosis and the expression of related proteins. We determined tumor growth in xenograft nude mice. Bioinformatic analysis, protein coimmunoprecipitation (Co-IP), luciferase assay, cell experiments were performed to reveal the relationship of Celastrol/KAT2B/RBPJ/MCM4 in UCEC.

**Results:**

Treatment of Celastrol inhibited cell viability in a dose-dependent manner, and caused cell cycle arrest, accompanied by the downregulation of CDK2 and cyclin E expression and the upregulation of p21. Celastrol treatment resulted DNA damage and apoptosis in cultured cells, as demonstrated by increased number of TUNEL-positive cells, activity of caspase-3 and expression of cleaved-caspase-9, cleaved PARP1 and γ-H2AX. In xenograft nude mice, Celastrol also repressed tumor growth. Furthermore, lysine acetyltransferase KAT2B was a putative target of Celastrol, and its expression was upregulated by Celastrol in vitro and in vivo. Overexpression of KAT2B in UCEC inhibited cell proliferation and increased DNA damage and apoptosis. KAT2B knockdown overcame the anti-proliferative and pro-apoptotic roles of Celastrol. Moreover, Co-IP demonstrated that KAT2B bound to RBPJ, a transcriptional repressor, and increased the acetylation of RBPJ. RBPJ could bind to the MCM4 promoter to suppress the luciferase activity. Further functional analysis revealed that the functions of KAT2B in UCEC cell proliferation, DNA damage and apoptosis were mediated by MCM4, and Celastrol enhanced RBPJ acetylation and reduced MCM4 expression.

**Conclusions:**

These results underscore that Celastrol is a promising anti-cancer agent in UCEC with preferential anti-proliferative, pro-apoptotic and DNA damage effects through the KAT2B/RBPJ/MCM4 axis, and KAT2B is a promising therapeutic target for UCEC.

**Graphical abstract:**

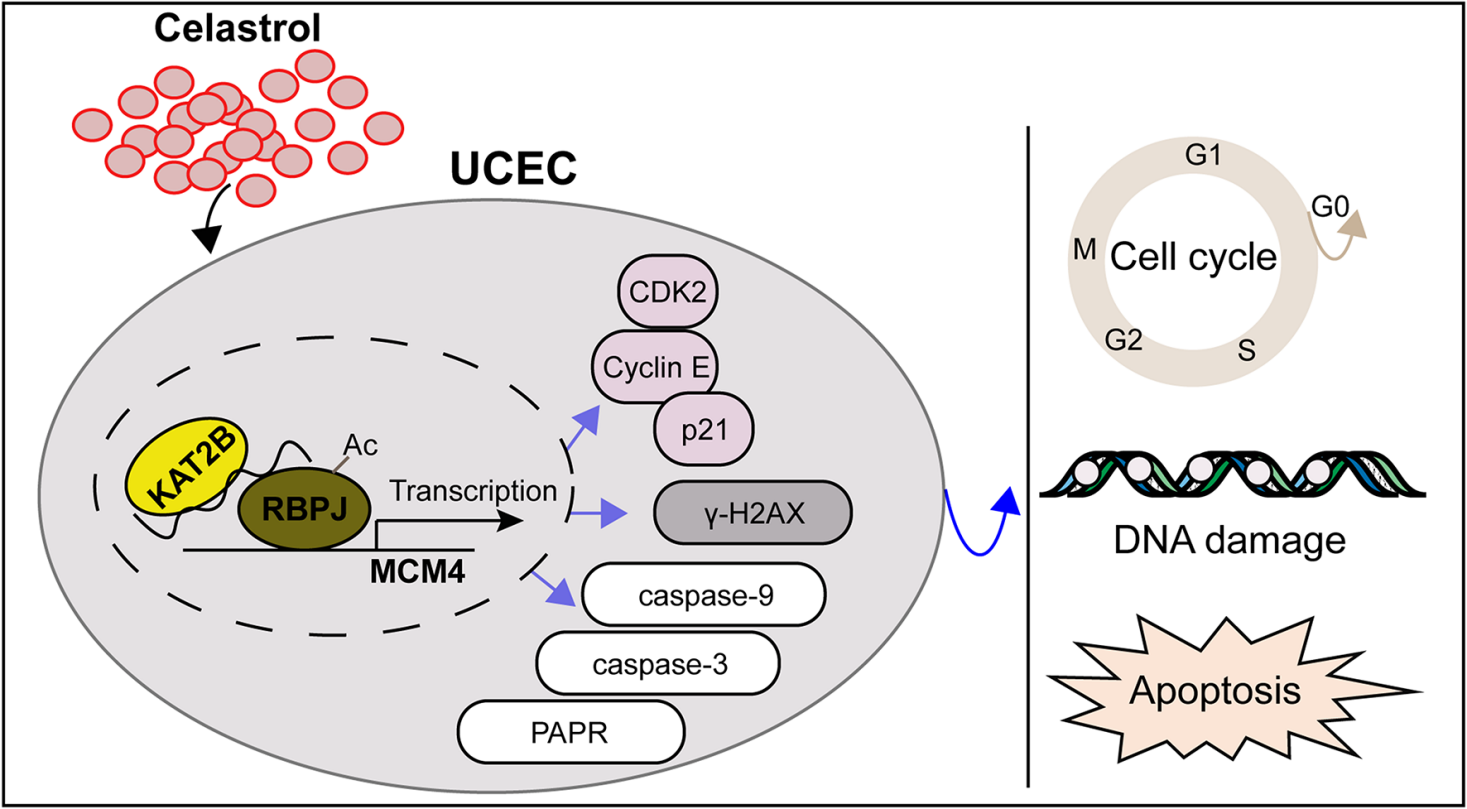

**Supplementary Information:**

The online version contains supplementary material available at 10.1186/s10020-025-01082-z.

## Introduction

Uterine corpus endometrial carcinoma (UCEC) is one of the most common malignant tumors in the female reproductive tract, which originated from the endometrial glands. The vast majority of UCECs are histologically adenocarcinomas (Abu-Rustum et al. 2023). Currently, surgery, chemotherapy and radiotherapy are the standard clinical treatment for UCEC patients. The lack of effective therapeutic strategies in many UCEC patients results in low 5-year survival rates (Hanley et al. 2017; Yagi and Ueda 2022). Therefore, the development of more efficient therapies for UCEC patients is urgently required.

Celastrol is a natural bioactive ingredient extracted from the Celastraceae plant family including Tripterygium wilfordii and Celastrus orbiculatus. Accumulating evidence shows that Celastrol can act as a good anti-carcinogenic agent due to its strong anti-cancer activity in many cancers, such as gastric cancer, colorectal cancer and lung cancer (Chen et al. 2020, 2022; Wang et al. 2023; Xu et al. 2023a). However, the role of Celastrol in UCEC remains unknown. It has been indicated that Celastrol and histone deacetylase inhibitors can synergize to influence tumor growth, implying that acetylation appears to play a key role in the anti-tumor efficacy of Celastrol (Chen et al. 2022). Lysine acetyltransferase 2B (KAT2B) is a known histone acetyltransferase epigenetic factor, which is involved in the proliferation and DNA damage of cancer cells (Chen and Allgayer 2023; Ma et al. 2024). KAT2B can increase the acetylation level of Fascin in to suppress tumor growth in esophageal cancer (Cheng et al. 2021). KAT2B decreases the acetylation of BRCA2 to enhance the sensitivity of cancer cells in colorectal cancer (Chen and Allgayer 2023). It is largely unknown whether Celastrol exerts function through modulating KAT2B in UCEC.

Additionally, KAT2B directly interacts with transcription factors, catalyzing the acetylation of non-histone proteins, and results in increased transcriptional activity (Ghosh et al. 2018; Song et al. 2002). Recombination signal-binding protein J (RBPJ) is a transcription factor that can serve as a transcriptional activator but also play a transcriptional repressive effect on different target genes (Xu et al. 2017). It has been proved that RBPJ blocks the movability of endometrial carcinoma cells (Xiao et al. 2019). Moreover, mini chromosome maintenance protein 4 (MCM4) promotes tumor growth in UCEC (Pei et al. 2022). Celastrol treatment induces the downregulation of MCM4 in cancer cells (Youns and Askoura 2021). Thus, RBPJ and MCM4 may also be implicated in Celastrol’s function in UCEC.

In the present study, we found that Celastrol facilitated cell apoptosis and DNA damage and inhibited tumor growth in UCEC. Mechanistically, Celastrol treatment upregulated KAT2B expression to increase the acetylation of RBPJ, thus inhibiting the transcription of MCM4 in UCEC. This study provides an evidence that Celastrol may be a candidate agent for new anti-cancer drugs in UCEC.

## Materials and methods

### Database construction and molecular docking

The chemical structure of Celastrol was obtained using the *PubChem* database (https://www.ncbi.nlm.nih.gov/pccompound/). *SwissTargetPrediction* database was applied to predict the target proteins of Celastrol. Gene ontology (GO) enrichment analysis was conducted on the common targets using the *STRING* database (https://string-db.org/). The molecular docking of Celastrol was performed with KAT2B (PDB code 4NSQ) using AutodockVina 1.2.2 software (Trott and Olson 2010). KAT2B, RBPJ and MCM4 expression in UCEC samples was analyzed using *Ualcan* (https://ualcan.path.uab.edu/). The *JASPAR* (http://jaspar.genereg.net/) indicated the binding motifs of RBPJ. The *GEPIA* database (http://gepia.cancer-pku.cn/) was utilized to analyze the gene correlation in UCEC samples.

### Cell culture and treatment

**Fig. 1:**
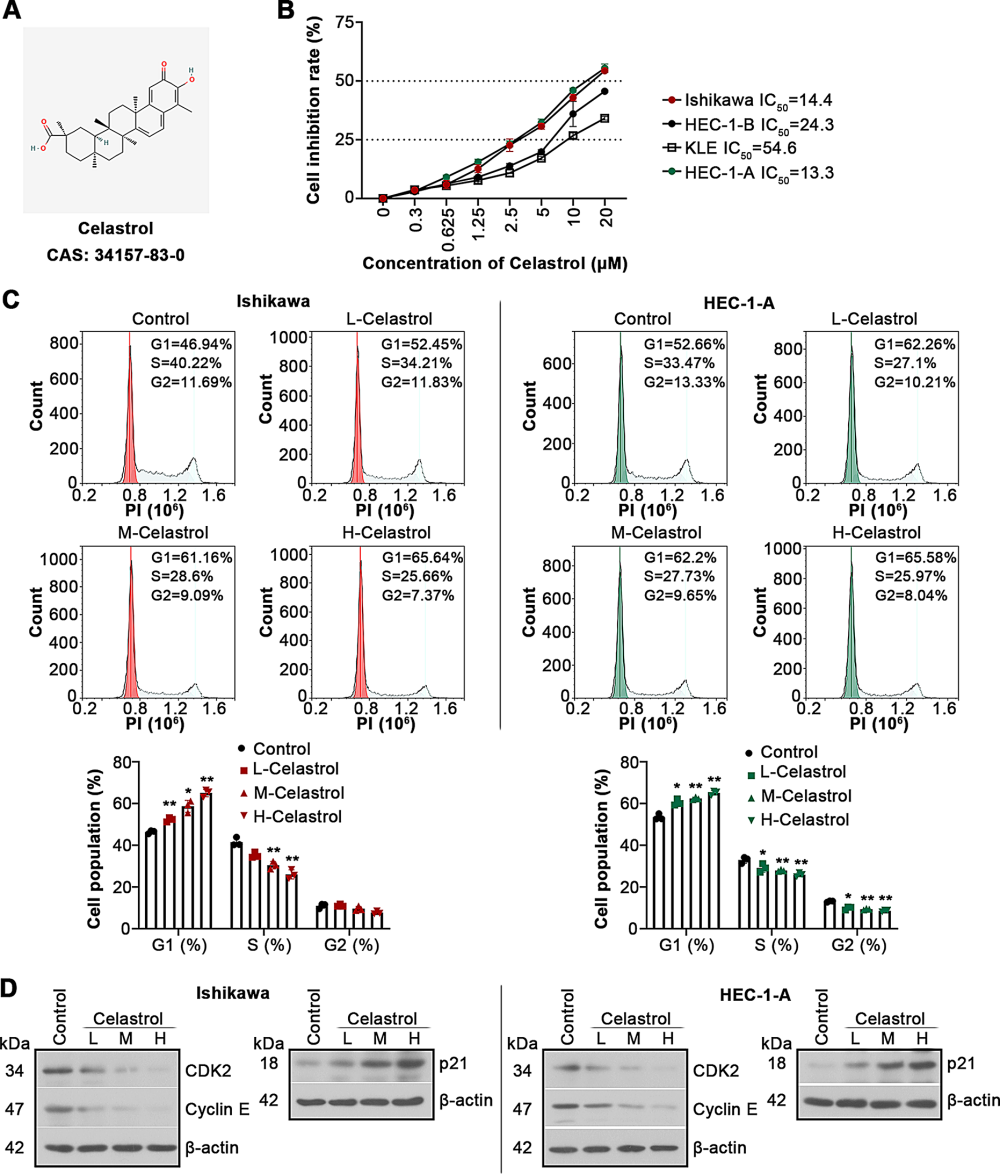
Celastrol suppresses cell proliferation in UCEC. Four UCEC cell lines Ishikawa, HEC-1-B, KLE and HEC-1-A were applied and treated with different concentrations of Celastrol (0, 0.3, 0.625, 1.25, 2.5, 5, 10 or 20 µM) for 24 h. (**A**) The chemical structure of Celastrol. (**B**) CCK8 assay tested the cell viability, and cell inhibition rate (CI) was calculated. (**C**) Ishikawa and HEC-1-A cells were treated with 2.5, 5 or 10 µM of Celastrol (L-Celastrol, M-Celastrol or H-Celastrol) and cultured for 24 h. The distribution of cell cycle was evaluated by flow cytometry analysis. (**D**) Cell cycle-associated protein expression levels were measured by western blotting assays. All the values are mean ± SD. ∗*P* < 0.05, ∗∗*P* < 0.01 vs. the control group

The human endometrial cancer cell lines Ishikawa, HEC-1-B, KLE and HEC-1-A were obtained from *iCell* Bioscience Inc (Shanghai, China). Ishikawa and HEC-1-B cells were cultured in MEM (*Solaibao*, Beijing, China), KLE cells were grown in DMEM/F12 (*Biosharp*, Hefei, China) and HEC-1-A cells were maintained in McCoy’s 5 A medium (*Servicebio*, Wuhan, China). All media contained 10% fetal bovine serum (FBS). The chemical structure formula of Celastrol was shown in Fig. 1A. Celastrol was purchased from *Macklin* Inc. (Shanghai, China). For cell treatment, cells were treated with different concentrations of Celastrol (0 µM, 0.3 µM, 0.625 µM, 1.25 µM, 2.5 µM, 5 µM, 10 µM, 20 µM) for 24 h. For lentivirus infection, Ishikawa and HEC-1-A cells were infected with lentiviruses expressing KAT2B overexpression plasmid or KAT2B shRNA, or MCM4 overexpression plasmid. At the indicated times, the cells were harvested for further experiments.

### Cell counting Kit-8 (CCK8) assay

Cell viability was assessed using CCK8 assay (*Source Leaf* Biological Technology Co., Ltd, Shanghai, China). The cells were seeded in 96-well plates (5 × 10^3^ cells/well) and cultured overnight in an incubator (37℃, 5% CO_2_). Then, 10 µl of CCK8 solution was added in each well and incubated for 1 h in the incubator. The OD value at 450 nm was measured using a microplate reader (*BioTek*, Winooski, VT, USA). The half maximal inhibitory concentration (IC_50_) of Celastrol was calculated by fitting the curve.

### Flow cytometric analysis

After centrifugation (150 *g*) for 5 min, the cells were collected and fixed in ice-cold 70% alcohol overnight. Then 40 µl PBS and 10 µl RNase A solution (50×) were added and the cells were incubated at 37℃ for 30 min. The cells were stained with 500 µl PI working solution (*Source Leaf* Biological Technology Co., Ltd, Shanghai, China) for 30 min at 4℃ in the dark, followed by flow cytometric analysis for cell cycle.

### Western blotting

The cells were lysed by cell lysates (*Beyotime*, Shanghai, China) for total protein extraction. After quantification using BCA Protein Assay Kit (*Beyotime*, Shanghai, China), equal amounts of protein were loaded onto SDS-PAGE gels. The proteins were transferred onto PVDF membranes (*Abcam*, Cambridge, UK), and the membranes were then blocked with blocking solution (*Beyotime*, Shanghai, China). Immunoblotting was performed using the following primary antibodies: anti-KAT2B (*Proteintech*, Wuhan, China), anti-CDK2, anti-Cyclin E, anti-p21, anti-cleaved-PARP1, anti-γ-H2AX, anti-pan acetyl-lysine, anti-MCM4 (*Zenbio*, Chengdu, China), and anti-cleaved-caspase-9 (*CST*, Boston, USA). The incubation of the primary antibody was performed overnight at 4℃ and that of the secondary antibody (anti-rabbit IgG-HRP) was 45 min at 37℃. β-actin was selected as the loading control. The enhanced chemiluminescence (ECL) reagent (*Beyotime*, Shanghai, China) was used to visualize the protein bands and the photos were taken in the darkroom.

### Protein immunoprecipitation (Co-IP)

Total protein was prepared and quantified, and immunoprecipitation was performed to verify protein-protein interaction. The centrifuged cell lysates were incubated with the indicated antibody, and the affinity-purified antibody was added and incubated for 120 min at room temperature. After washing with lysis buffer (*Beyotime*, Shanghai, China), western blotting was performed for analyzing anti-KAT2B, anti-RBPJ (*Proteintech*, Wuhan, China), or anti-pan acetyl-lysine immunoreactivity.

### TUNEL apoptosis assay

Cell apoptosis was determined using a TUNEL Apoptosis Assay Kit (*Roche*, Basel, Switzerland). The cells were treated for 15 min with 0.1% Triton X–100 (*Beyotime*, Shanghai, China) for permeabilization. After washing, the TUNEL working solution was prepared and added onto the cells. The cells were incubated for 60 min at 37℃ in the dark. DAPI staining solution (*Aladdin*, Shanghai, China) was used for color development. The staining result was observed using a fluorescence microscope (*Olympus*, Japan).

### Caspase-3 activity

Caspase-3 activity in cells was evaluated with a Caspase-3 Activity Assay Kit (*Beyotime*, Shanghai, China). All experimental operations were carried out according to the manufacturer’s instructions. The prepared samples were mixed with the assay buffer, cell lysate and Ac-DEVD-pNA, and incubated for 60 min at 37℃. The absorbance at 405 nm was measured using a microplate reader.

### Comet assay

The comet assay was performed according to the *Wanleibio* Comet Assay Kit instruction (Shengyang, China). The cells were mixed with 2% low-melting point agarose (1: 1), and then the mixture was spread on a culture dish with 0.5% normal melting point agarose. The dish was placed at 4℃ to solidify, and the slides after lysis were immersed for 45 min in precooled electrophoresis buffer. Following 60 min of electrophoresis (40 V), the slides were stained with propidium iodide (PI) staining solution and photographed under a fluorescence microscope. The extent of DNA damage was assessed based on the percentage of DNA in the comet tail as described (Wang et al. 2019).

### Immunofluorescence (IF)

The cells were fixed for 15 min with 4% paraformaldehyde and permeabilized for 30 min with 0.1% Triton X-100 (*Beyotime*, Shanghai, China). After blocked for 15 min with 1% BSA (*Sangon*, Shanghai, China), the cells were incubated overnight with primary antibody (anti-γ-H2AX, *Zenbio*, Chengdu, China) at 4℃. The secondary antibody was then added and the cells were incubated 60 min at room temperature. Nuclei were stained with DAPI. The images were captured using a fluorescence microscope, and the γ-H2AX-positive cells was quantified by manual counting.

### I*n vivo* experiments

Healthy female Balb/c nude mice aged 5 weeks were used in our study. I*n vivo* experimental protocols were approved by the General Hospital of Northern Theater Command Institutional Animal Care and Use Committee. Ishikawa cells were subcutaneously injected into each mouse at a density of 1 × 10^6^ cells. Once the tumors were visible, the mice were intraperitoneally injected with Celastrol at the dosage of 1 mg/kg or 2 mg/kg. Celastrol was administrated once daily for 5 days, with 2-day intervals to the next week. Then, tumor volume and body weight of mice were monitored every four days. Four weeks later, the mice were euthanized and the xenograft tumors were collected for further analysis.

### Immunohistochemistry (IHC)

The 5-µm-thick sections from tumor tissues were embedded in paraffin, dewaxed and then subjected to antigen repair. After incubated with the anti-Ki-67 (*Affinity*, Changzhou, China) overnight at 4℃, the sections were incubated with the secondary antibody. Visualization was achieved by DAB color-developing solution (*Solarbio*, Beijing, China), and counterstaining was done using Hematoxylin (*Solarbio*, Beijing, China). The staining was observed under a DP73 microscope.

### Real-time quantitative PCR (RT-qPCR)

Total RNA from the cells was extracted using TRIpure (*BioTeke*, Beijing, China), and the first cDNA strand was synthesized using All-in-One First-Strand SuperMix (*Magen*, Guangzhou, China). KAT2B and MCM4 mRNA expression levels were determined using the RT-qPCR assay with 2×Taq PCR MasterMix and SYBR Green (*Solarbio*, Beijing, China). The experimental protocols were performed according to the instructions of the manufacturers. The following primers were utilized for RT-qPCR assay: KAT2B, forward, 5’-GTTGAAGGCTCTTTGGA-3’ and reverse, 5’-AGTTGATGCGGTTTAGG-3’; MCM4, forward, 5’-TAGTGGCAAGTGAGCAG-3’ and reverse, 5’-CCAGAGGGTCAATAAAA-3’; β-actin, forward, 5’-GGCACCCAGCACAATGAA-3’ and reverse, 5’-TAGAAGCATTTGCGGTGG-3’.

### Luciferase assay

Human 293T cells were grown in DMEM (*Servicebio*, Wuhan, China) supplemented with 10% FBS. The cells were transfected with the plasmids using Lipofectamine 8000 (*Beyotime*, Shanghai, China) following the protocol. After 48 h of transfection, the luciferase activity was measured by a Luciferase Assay Kit (*KeyGen*, Nanjing, China). Renilla plasmid was used as the internal control.

### Statistical analysis

The results were represented as means and standard deviation (SD). Statistical analysis was performed using GraphPad Prism software 8.0. Difference between two groups was evaluated by Student’s t-test, while comparison between multiple groups was analyzed using one-way analysis of variance (ANOVA). Two-way ANOVA was utilized for comparison between multiple groups at different time points. P-value < 0.05 was considered statistically significant.

## Results

### Celastrol suppresses cell proliferation in UCEC

UCEC cells were treated with increasing concentrations of Celastrol for 24 h, and CCK8 assay was utilized to detect the cell viability. It was showed that Celastrol inhibited cell viability in a dose-dependent manner, and IC_50_ values of Celastrol in Ishikawa and HEC-1-A cells were close (Fig. 1B). The concentrations used were 2.5, 5 and 10 µM in the subsequent experiments. Flow cytometry analysis for cell cycle distribution showed that Celastrol resulted in the cell-cycle arrest in the G1 phase (Fig. 1C). Celastrol also exhibited an inhibition in CDK2 and cyclin E but an induction in p21 in Ishikawa and HEC-1-A cells (Fig. 1D). These results show that Celastrol suppresses cell proliferation in UCEC.

### Celastrol facilitates cell apoptosis and DNA damage in UCEC

**Fig. 2:**
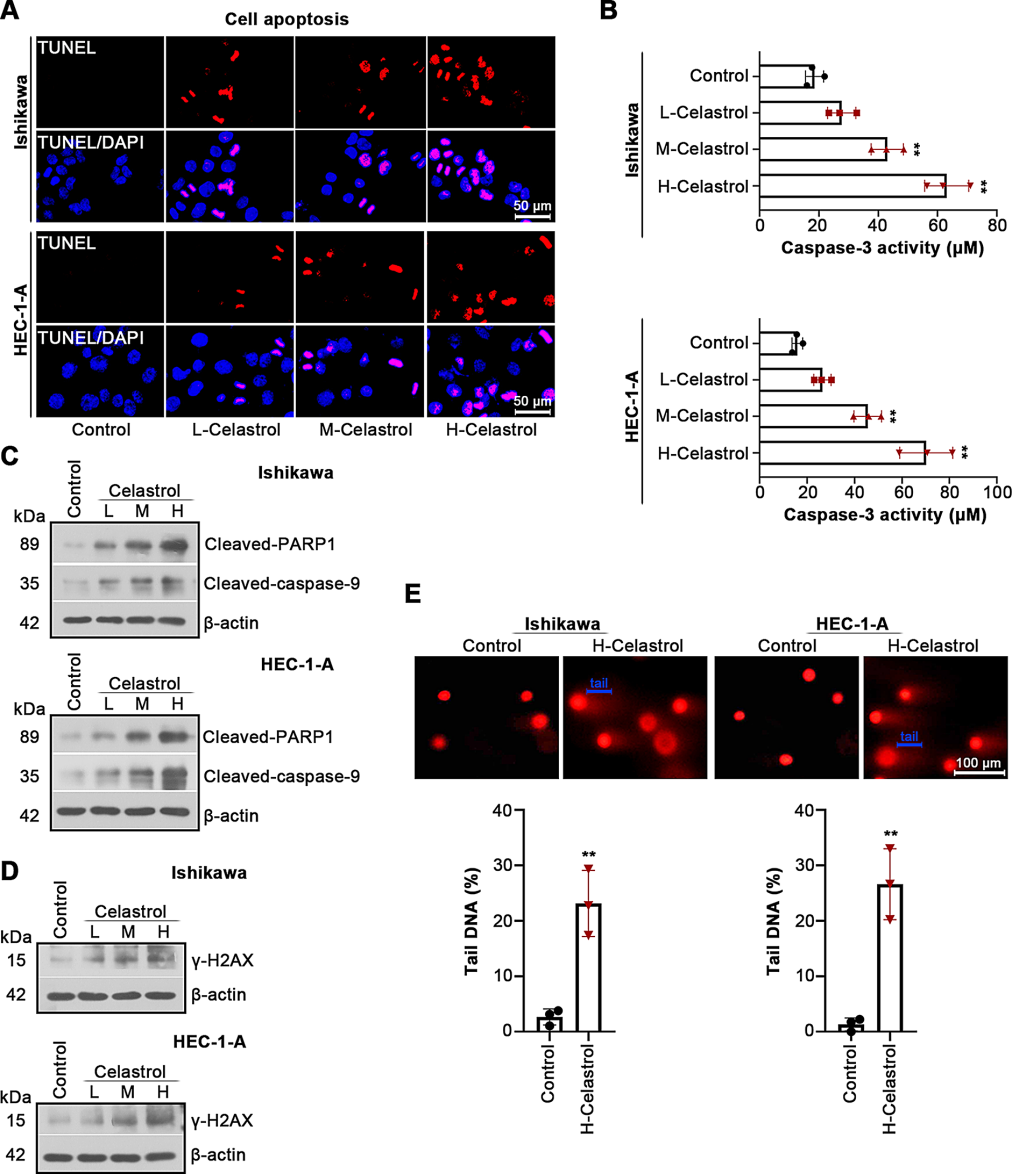
Celastrol induces DNA damage and apoptosis of UCEC cells. (**A**) TUNEL staining was conducted for evaluating cell apoptosis. Scale bars, 50 μm. (**B**) Caspase-3 activity in cells treated for 24 h with L-Celastrol, M-Celastrol or H-Celastrol. (**C**, **D**) Relative protein expression of cleaved-caspase-9, cleaved-PARP1 and γ-H2AX was detected by western blotting. (**E**) Representative pictures of comets, and the tails of comets were indicated. Scale bars, 100 μm. All the values are mean ± SD. ∗∗*P* < 0.01 vs. the control group

We investigated the effects of Celastrol on cell apoptosis and DNA damage in UCEC. As shown in Fig. 2A, TUNEL staining demonstrated that the numbers of apoptotic cells at 24 h were increased by Celastrol treatment in dose-dependent manner. The cells treated with Celastrol showed higher caspase-3 activity, cleaved-caspase-9 and cleaved PARP1 expression levels than the control (Fig. 2B, C). Western blotting analysis of γ-H2AX displayed that Celastrol resulted in elevated γ-H2AX level in UCEC cells (Fig. 2D). The comet assay revealed severe DNA damage caused by Celastrol (Fig. 2E). These findings suggest that Celastrol contributes to apoptosis and DNA damage in cultured UCEC cells.

### Celastrol suppresses tumor growth of UCEC

**Fig. 3:**
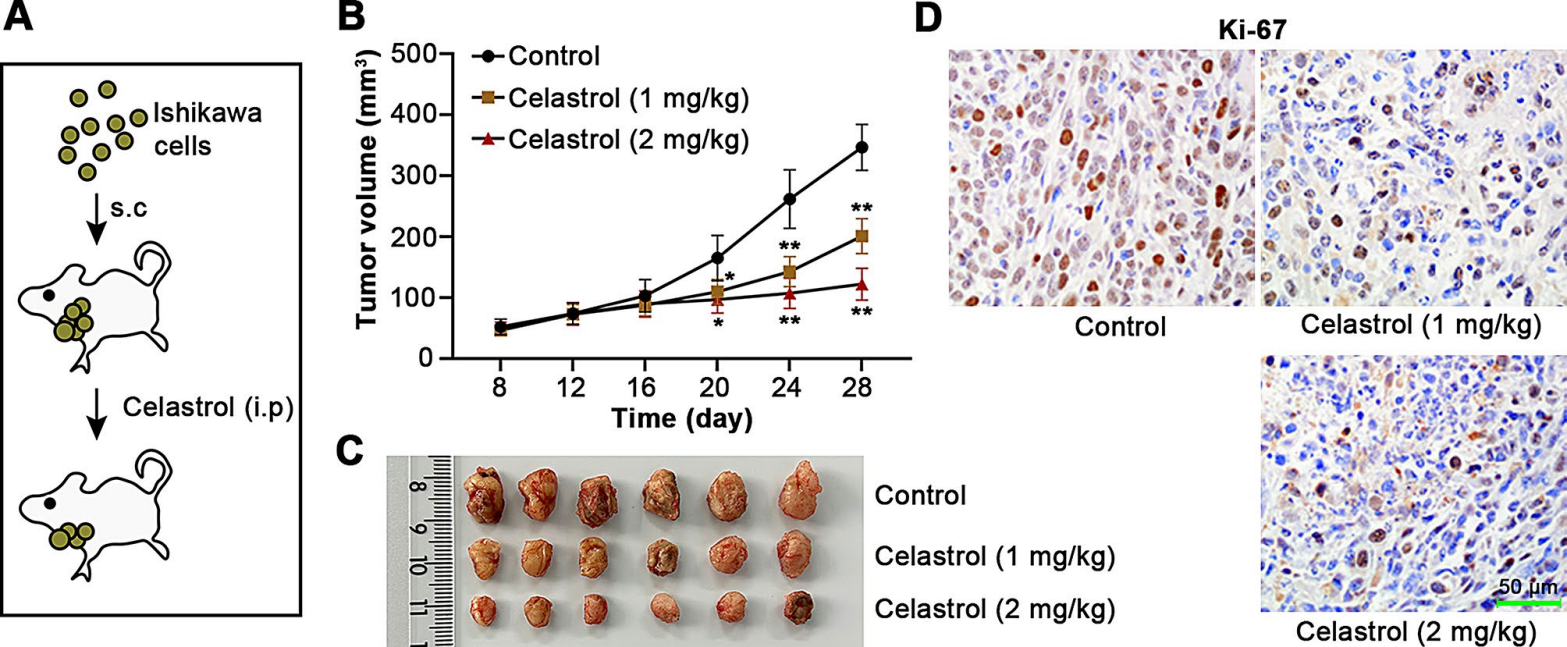
Celastrol inhibits tumor growth in xenograft mice. (**A**) The procedures of in vivo experiments. s.c, subcutaneous injection; i.p, intraperitoneal injection. (**B**) Tumor volumes were recorded every 4 days. (**C**) After 4 weeks of Celastrol administration, the tumors were photographed. (**D**) Immunohistochemistry (IHC) staining was performed to detect Ki-67 level in tumors. Scale bars, 50 μm. All the values are mean ± SD. ∗*P* < 0.05, ∗∗*P* < 0.01 vs. the control group

I*n vivo* experiments were conducted according to the procedures shown in Fig. 3A. Compared with the control group, treatments of mice with Celastrol at 1 and 2 mg/kg showed obviously suppressed tumor volume (Fig. 3B). At the end of the experiment, the tumors were photographed, as exhibited in Fig. 3C. According to Ki-67 staining results, treatment of Celastrol decreased the expression of Ki-67 protein in tumor tissues (Fig. 3D). The above results demonstrated that Celastrol inhibits tumor growth in a xenograft mouse model.

### KAT2B represents a potential target of Celastrol in UCEC

**Fig. 4:**
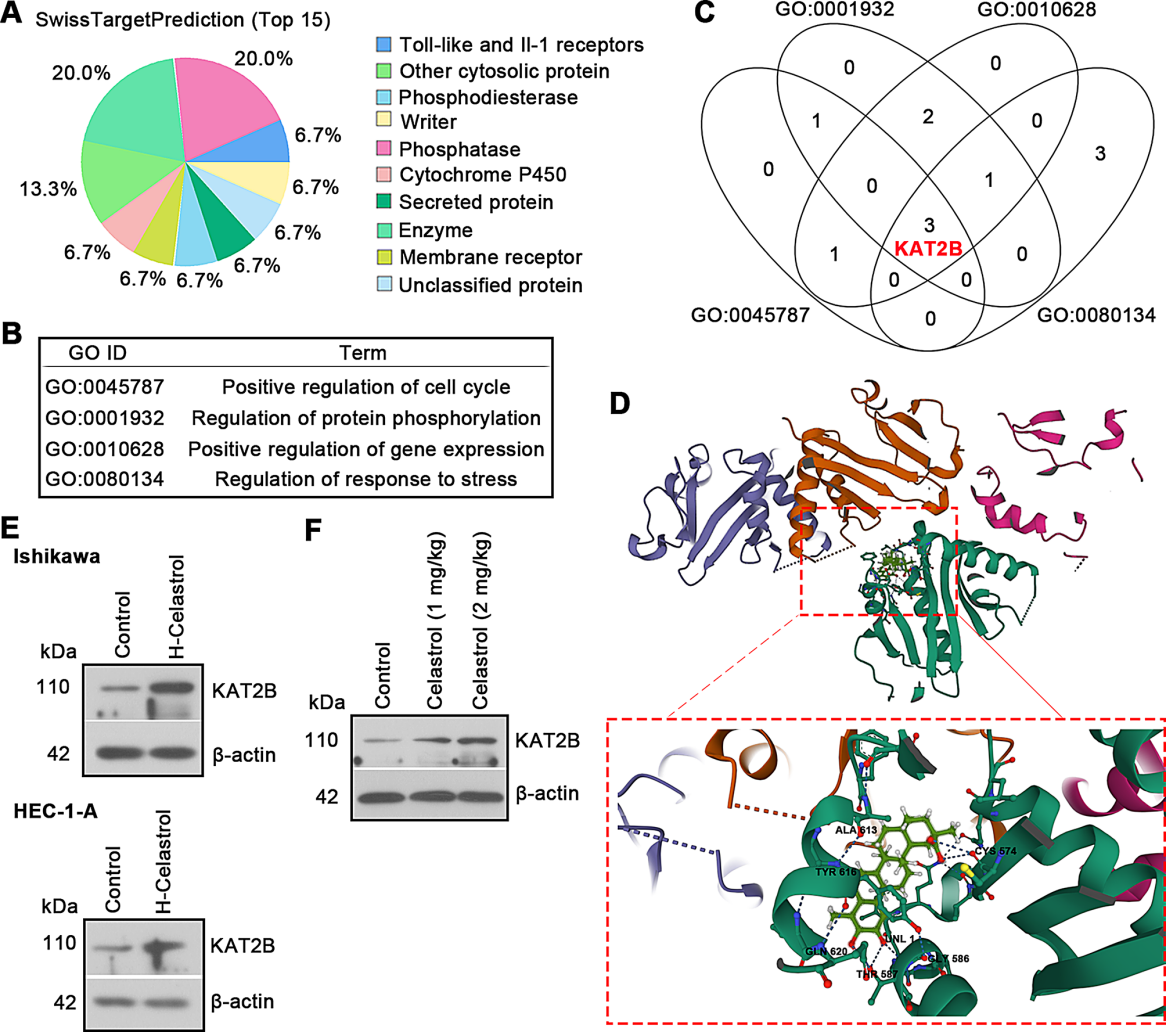
Celastrol may bind to KAT2B and increase its expression. (**A**) The distribution map of top 15 target proteins of Celastrol based on *SwissTargetPrediction* database. (**B**) The gene ontology (GO) analysis of the targets via *STRING*. (**C**) Venn diagram displayed the common targets involved in the above biological process. (**D**) The possible interaction between Celastrol and KAT2B by molecular docking analysis. (**E**) In cultured UCEC cells, KAT2B expression was decreased in response to Celastrol. (**F**) Relative KAT2B expression in tumors of Celastrol-treated xenograft mice

To uncover the potential mechanisms mediating the role of Celastrol in UCEC, the *SwissTargetPrediction* was applied to show the possible targets. Figure 4A displayed the distribution range of top 15 target proteins. GO analysis revealed that these targets were mainly enriched in the biological processes, including positive regulation of cell cycle, regulation of protein phosphorylation, positive regulation of gene expression and regulation of response to stress (Fig. 4B). Special attention was paid to the targets that involved in the above biological processes, and a total of 3 overlapped targets were identified. Based on a thorough literature research, KAT2B, a histone acetyltransferase epigenetic factor was concerned (Fig. 4C). Based on molecular docking analysis, we observed that Celastrol could dock with the protein KAT2B (PDB: 4NSQ), with a low binding energy of -9.367 kcal/mol, suggesting highly stable binding (Fig. 4D). Following 24 h of Celastrol treatment, the expression level of KAT2B protein was significantly upregulated in cultured UCEC cells (Fig. 4E). Celastrol treatment in tumor tissues increased KAT2B expression, as shown in Fig. 4F.

### KAT2B’s function in cell proliferation, apoptosis and DNA damage in UCEC

**Fig. 5:**
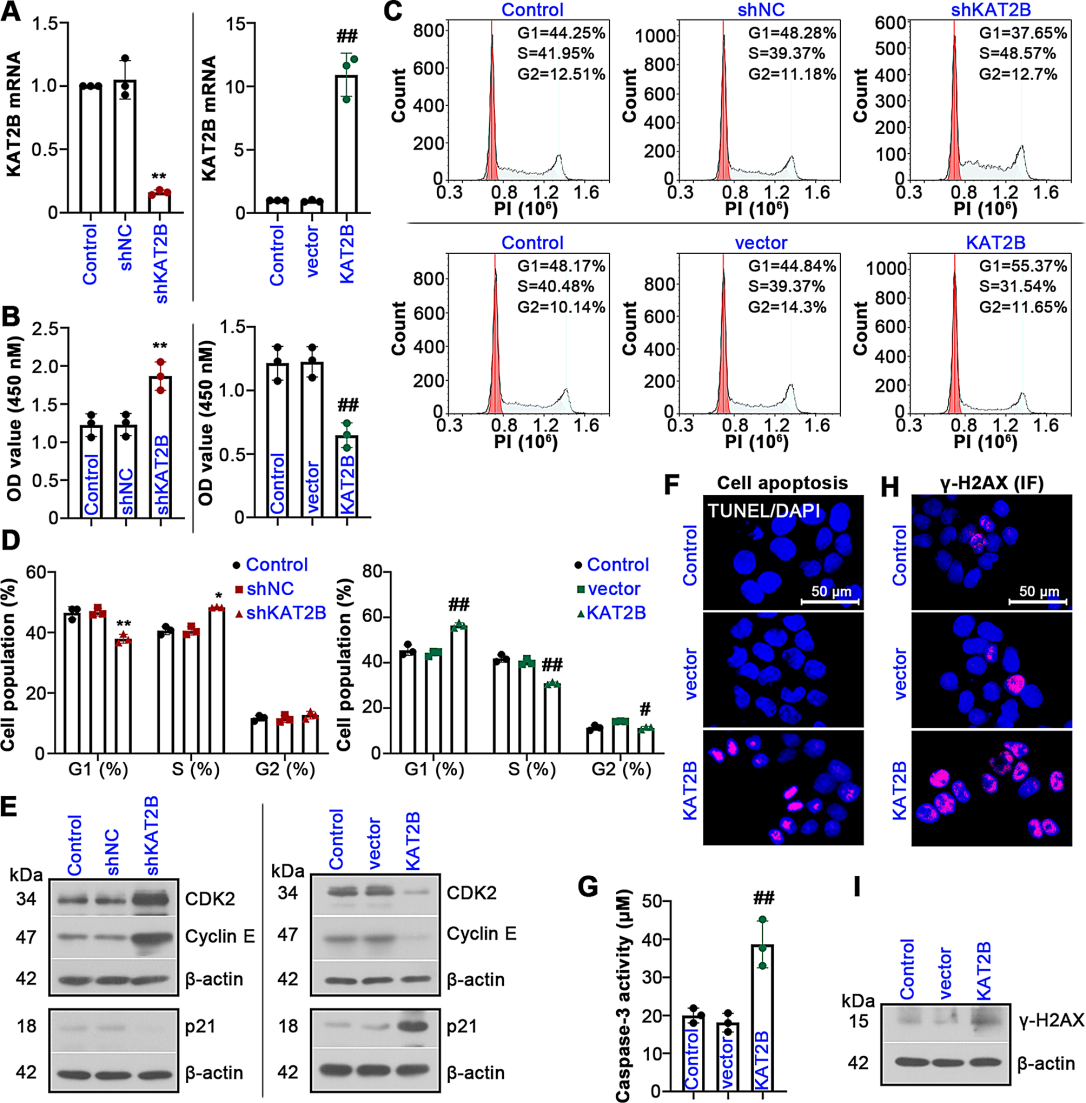
Effect of KAT2B on UCEC cell proliferation, DNA damage and apoptosis. (**A**) RT-qPCR results showing the knockdown and overexpression of KAT2B in UCEC cells. (**B**) The changes in cell viability by CCK8 assay. (**C**, **D**) The distribution of cell cycle. (**E**) Relative expression levels of CDK2, cyclin E and p21. (**F**) TUNEL staining showing the apoptotic cells. (**G**) Caspase-3 activity was detected. (**H**, **I**) Immunofluorescent (IF) staining and western blotting analysis of γ-H2AX. Scale bars, 50 μm. All the values are mean ± SD. ∗*P* < 0.05, ∗∗*P* < 0.01 vs. the shNC group. #*P* < 0.05, ##*P* < 0.01 vs. the vector group

As shown in Fig. S1A, *Ualcan* analysis revealed that the expression of KAT2B was downregulated in UCEC tissue samples. To explore the role of KAT2B in UCEC, the subsequent experiments were performed. RT-qPCR assay showed that KAT2B expression level in UCEC cells was knock downed by shKAT2B, while overexpressed by KAT2B (Fig. 5A). CCK8 assay revealed that KAT2B knockdown enhanced cell viability, while its overexpression inhibited cell viability (Fig. 5B). Flow cytometry analysis showed that KAT2B knockdown promoted cell cycle progression, whereas an inhibitory effect of overexpression of KAT2B on cell cycle entry was observed in UCEC cells (Fig. 5C, D). The expression levels of CDK2 and cyclin E were increased but p21 level was reduced in Lv-shKAT2B-infected cells, and the opposite results were observed in Lv-KAT2B-infected cells (Fig. 5E). Besides, due to downregulated KAT2B expression in UCEC, we hypothesized that KAT2B might be an anti-cancer gene in UCEC. As expected, our experiments demonstrated that KAT2B overexpression caused apoptosis of UCEC cells (Fig. 5F), accompanied by increased caspase-3 activity (Fig. 5G). The cells overexpressing KAT2B had higher γ-H2AX expression than the Lv-vector group, as validated by IF staining and western blotting analysis (Fig. 5H, I and Fig. S2A).

### KAT2B mediates Celastrol-induced cell apoptosis and DNA damage in UCEC

**Fig. 6:**
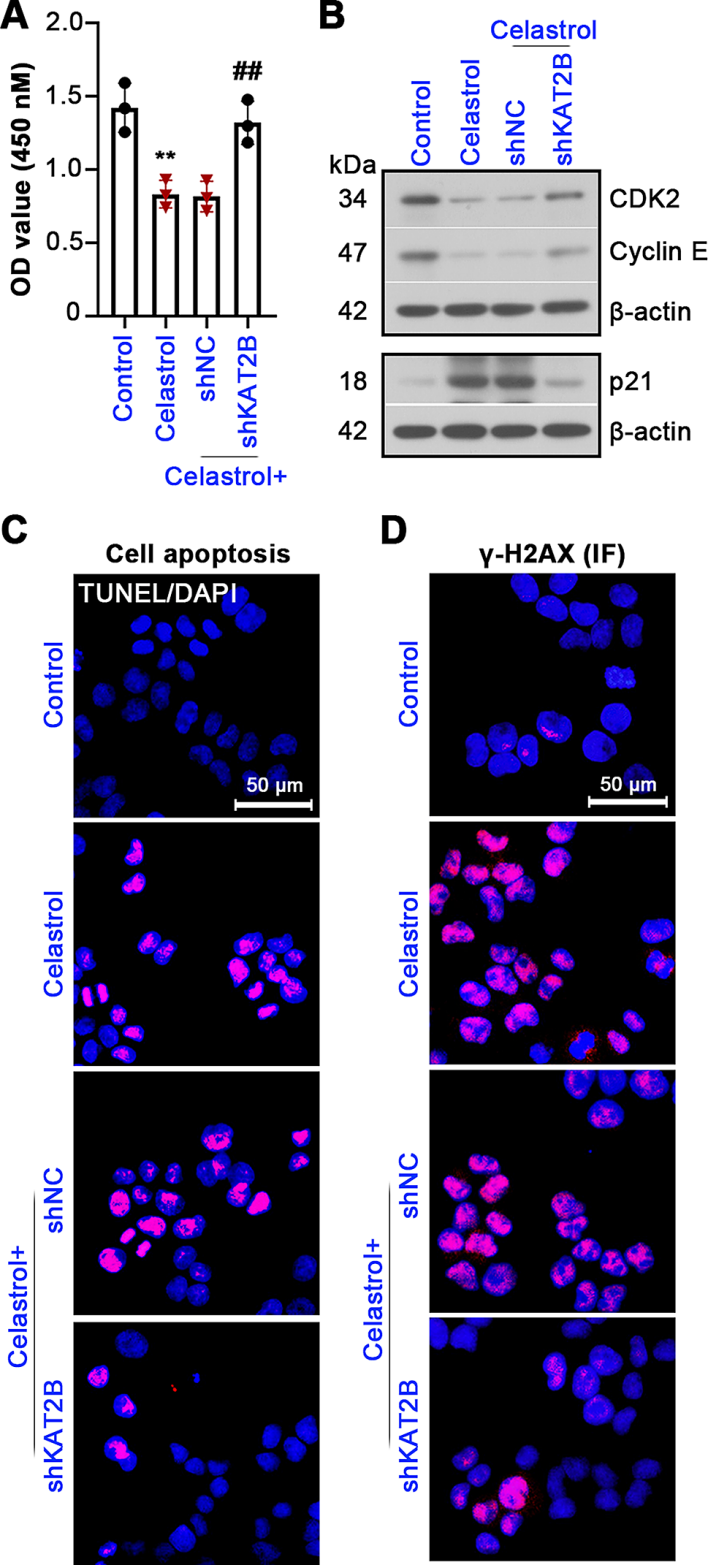
Celastrol functions in UCEC through KAT2B. (**A**) After infection of lentivirus expressing shKAT2B or shNC, Ishikawa cells were treated for 24 h with 10 µM of Celastrol. Cell viability was detected by CCK8 kit. (**B**) Expression of CDK2, cyclin E and p21 in cells was determined. (**C**) Cell apoptosis was analyzed using TUNEL staining. (**D**) Representative images of γ-H2AX IF staining. All the values are mean ± SD. ∗∗*P* < 0.01 vs. the control group. ##*P* < 0.01 vs. the Celastrol + shNC group

To confirm the functional link between Celastrol and KAT2B, the cells infected with Lv-shKAT2B or Lv-shNC were treated with Celastrol for 24 h. KAT2B knockdown in the Lv-shKAT2B group significantly abolished Celastrol-caused inhibition of cell viability (Fig. 6A). Compared with the Celastrol-treated Lv-shNC cells, elevated expression of CDK2 and cyclin E and reduced p21 were found in the Celastrol-treated Lv-shKAT2B cells (Fig. 6B). TUNEL staining uncovered that knockdown of KAT2B abrogated Celastrol-induced cell apoptosis, as shown in Fig. 6C. KAT2B knockdown in UCEC cells inhibited γ-H2AX expression induced by Celastrol (Fig. 6D and Fig. S2B). Collectively, KAT2B may mediate Celastrol’s function in UCEC cell proliferation, apoptosis and DNA damage.

### RBPJ and MCM4 are involved in KAT2B’s modulation in UCEC

**Fig. 7:**
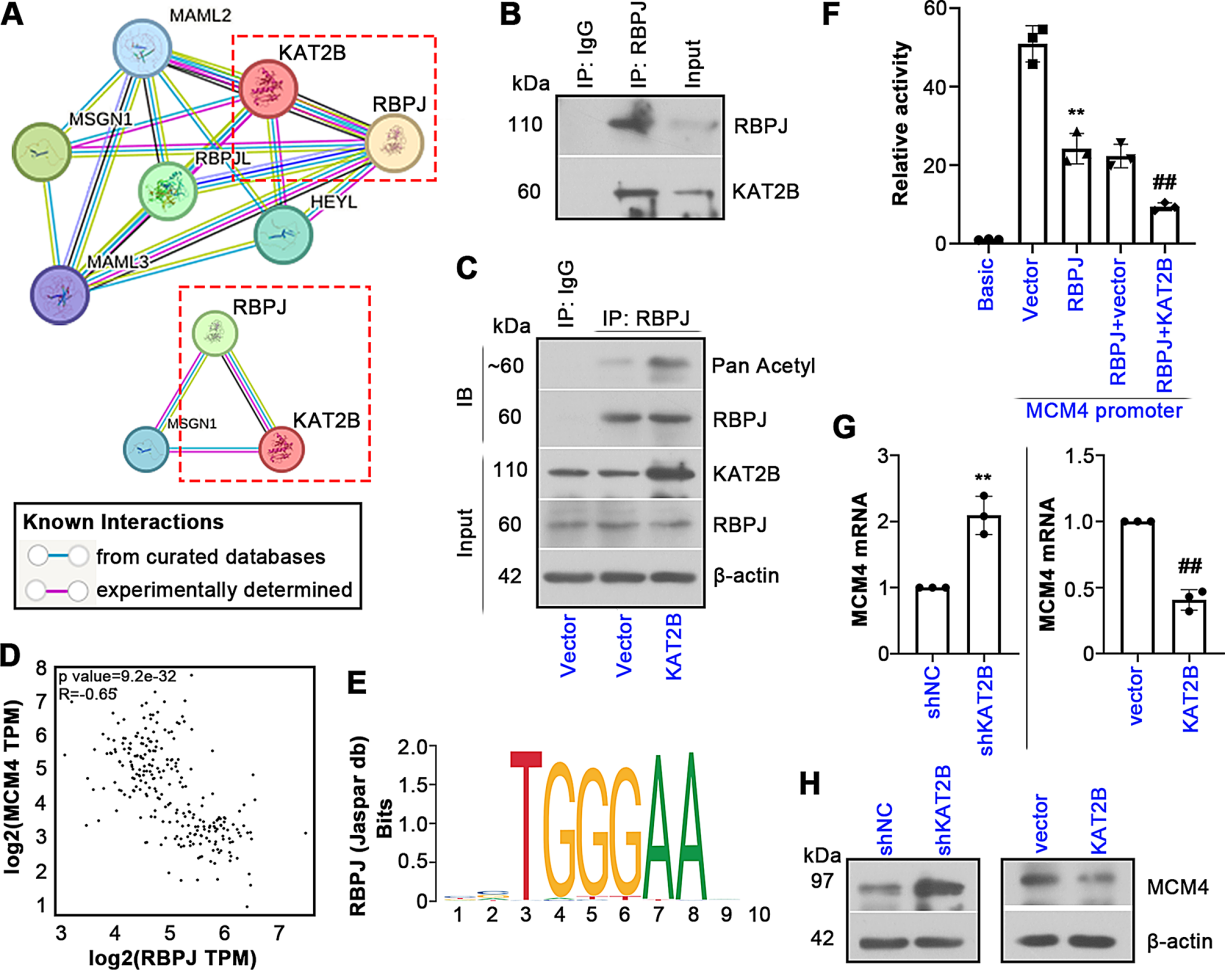
Relationship of KAT2B, RBPJ and MCM4. (**A**) The PPI network of KAT2B using *STRING*. (**B**) Protein coimmunoprecipitation (Co-IP) showed the association of KAT2B and RBPJ. (**C**) Co-IP assay was performed to determine the acetylation level of RBPJ. (**D**) Correlation of the expression of KAT2B and RBPJ in UCEC tumors using *GEPIA* analysis. (**E**) The binding motif of RBPJ was predicted by *JASPAR*. (**F**) Dual-luciferase assay was conducted to verify the relationship of KAT2B, RBPJ and MCM4. ∗∗*P* < 0.01 vs. the MCM4 + vector group. ##*P* < 0.01 vs. the MCM4 + RBPJ + vector group. (**G**, **H**) Relative expression of MCM4 in cells after KAT2B knockdown or overexpression. All the values are mean ± SD. ∗∗*P* < 0.01 vs. the shNC group. ##*P* < 0.01 vs. the vector group

We then clarified the molecular mechanism by which KAT2B influenced UCEC cell behavior. *STRING* database indicated the predicted binding proteins of KAT2B, as displayed in Fig. 7A. Among which, RBPJ has been reported to be closely correlated with UCEC (Feng and He 2023), which raised our attention. As shown in Fig. S1B, *Ualcan* analysis revealed the downregulated RBPJ in UCEC tissue samples. Protein immunoprecipitation confirmed the interaction of KAT2B with RBPJ in UCEC cells (Fig. 7B). I*n vitro* experiment revealed that KAT2B overexpression increased the acetylation of RBPJ (Fig. 7C). These results suggest that KAT2B can direct acetylate RBPJ in UCEC cells. In addition, Fig. S1C showed the upregulated MCM4 expression in UCEC tissue samples. *GEPIA* database showed that RBPJ had significant negative correlations with MCM4 in UCEC (Fig. 7D), indicating the involvement of MCM4 in KAT2B/RBPJ-mediated function in UCEC. Figure 7E displayed the binding motif of RBPJ predicted from *JASPAR* database. Luciferase assay demonstrated that RBPJ overexpression decreased the luciferase activity of MCM4 promoter, and KAT2B overexpression further inhibited it (Fig. 7F). RT-qPCR and western blotting assays verified that knockdown of KAT2B in UCEC cells induced the increases in MCM4 at both mRNA and protein levels, and KAT2B overexpression exerted the opposite effects (Fig. 7G, H). These results suggest that KAT2B enhances the transcriptional repressive ability of RBPJ on MCM4 in UCEC cells.

### MCM4 mediates KAT2B’s effect in UCEC cell proliferation, apoptosis and DNA damage

**Fig. 8:**
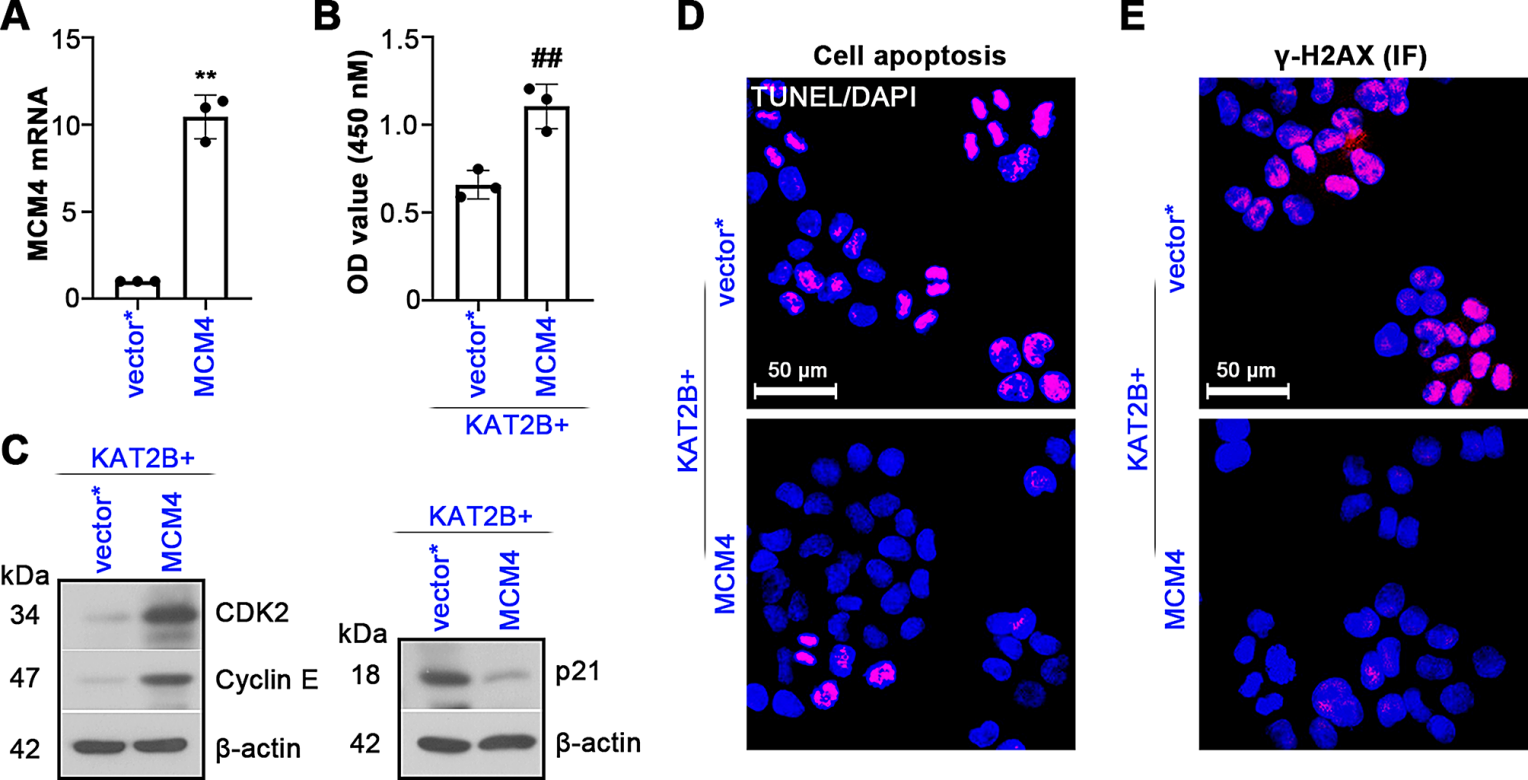
KAT2B’s function was mediated by MCM4. (**A**) RT-qPCR result demonstrated the overexpression of MCM4 in UCEC cells. (**B**) Cell viability was determined by CCK8 assay. (**C**) CDK2, cyclin E and p21 protein levels were showed. (**D**) TUNEL staining of UCEC cells. (**E**) IF staining of γ-H2AX in cells. All the values are mean ± SD. ∗∗*P* < 0.01 vs. the vector group. ##*P* < 0.01 vs. the KAT2B + vector group

To confirm whether MCM4 mediates the biological function of KAT2B in UCEC, overexpression of MCM4 in UCEC cells was achieved by Lv-MCM4, as displayed in Fig. 8A. CCK8 assay showed that MCM4 overexpression significantly increased the viability of cells overexpressing KAT2B (Fig. 8B). Western blotting assay showed that overexpression of MCM4 upregulated CDK2, cyclin E expression and downregulated p21 in KAT2B overexpressed cells (Fig. 8C). TUNEL staining revealed that MCM4 overexpression inhibited the increase in KAT2B-induced cell apoptosis (Fig. 8D). IF staining of γ-H2AX uncovered that the effect of KAT2B overexpression on γ-H2AX expression was reversed by overexpression of MCM4 (Fig. 8E and Fig. S2C).

### RBPJ and MCM4 are involved in Celastrol’s effect in vitro

**Fig. 9:**
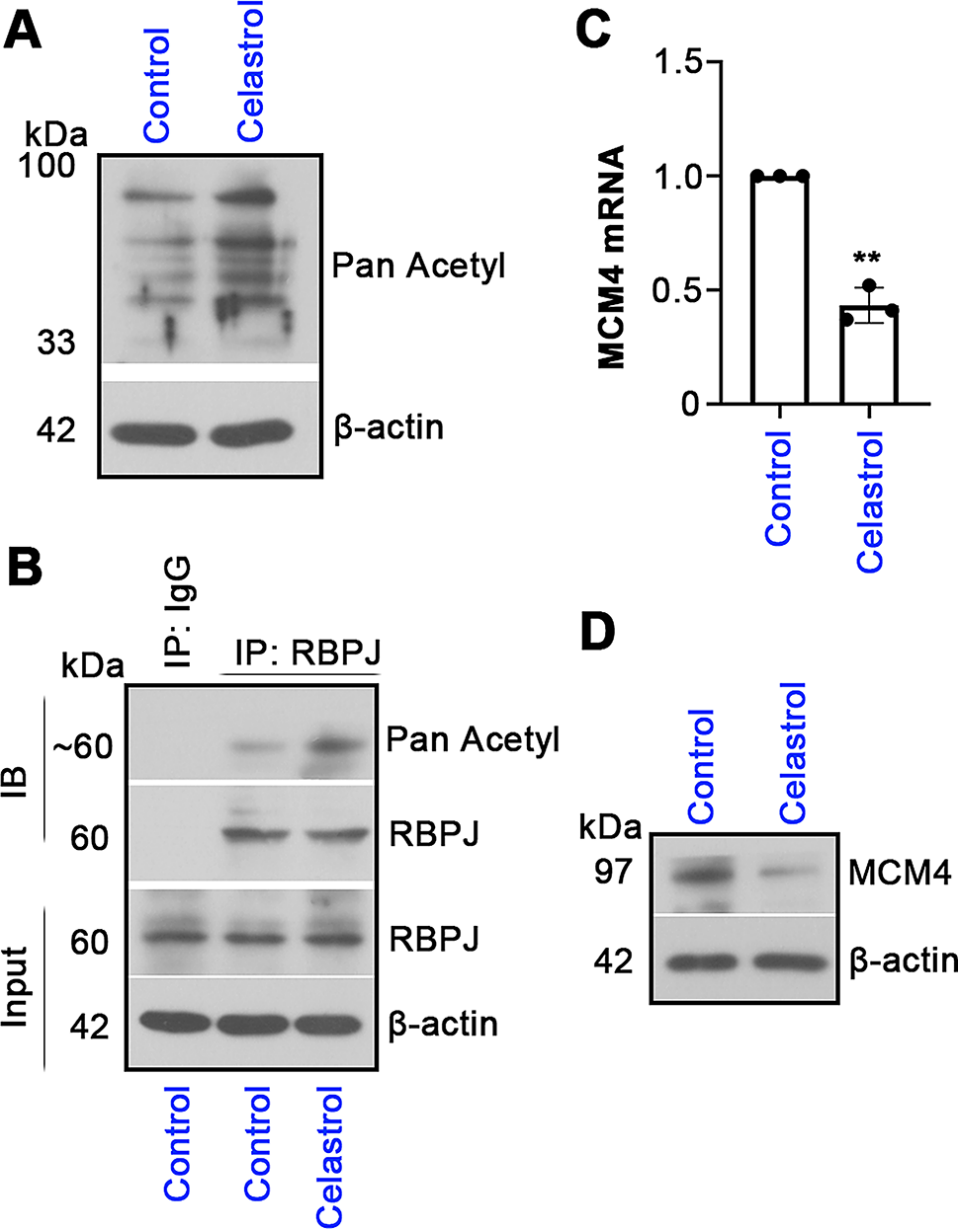
Celastrol affects RBPJ acetylation and MCM4 expression. (**A**) Co-IP assay was performed to detect the pan acetylation level in cells with or without Celastrol. (**B**) The acetylation level of RBPJ was determined by Co-IP. (**C**, **D**) The changes in MCM4 mRNA and protein expression levels in Celastrol-treated cells were measured by RT-qPCR and western blotting analysis. All the values are mean ± SD. ∗∗*P* < 0.01 vs. the control group

To verify whether the effect of Celastrol is linked to the regulation of RBPJ and MCM4, the following experiments were conducted. Figure 9A results showed that the level of pan-acetylation in UCEC cells was increased by Celastrol treatment. Further analysis found that treatment of Celastrol enhanced the acetylation of RBPJ (Fig. 8B). RT-qPCR and western blotting assays found that Celastrol suppressed the expression of MCM4 at mRNA and protein levels (Fig. 9C, D). These findings provide supportive data that Celastrol’s effect on UCEC cells is linked to the modulation of RBPJ acetylation and MCM4 expression.

## Discussion

In our study, we demonstrated that Celastrol effectively suppressed proliferation, facilitated apoptosis and DNA damage of UCEC cells and inhibited tumor growth in vivo. We elucidated a novel mechanism that Celastrol’s function in UCEC cells might be mediated by KAT2B/RBPJ/MCM4 axis. KAT2B expression was downregulated in UCEC tissue samples, and its overexpression resulted in increased apoptosis and DNA damage in UCEC cells, as accompanied by enhanced RBPJ acetylation and transcriptional inhibition of MCM4. As far as we know, this study is the first to prove that Celastrol exerts anti-cancer effects in UCEC and KAT2B acts as a potential target of Celastrol.

Celastrol as a natural pharmaceutical product has been evidenced to exert an anti-tumor efficacy in multiple human cancers (Chen et al. 2020, 2022; Wang et al. 2023; Xu et al. 2023a). Whether Celastrol performs the same effect in UCEC needs to be investigated. The endometrial cancer cell lines Ishikawa, HEC-1-B, KLE and HEC-1-A were selected in this study. The working concentrations of Celastrol were applied based on a previous study on Celastrol (Liu et al. 2021). CCK8 result indicated that IC_50_ values of Celastrol in Ishikawa and HEC-1-A cells were close, thus Ishikawa and HEC-1-A cells were selected for further studies. We noticed very high IC_50_ value of Celastrol in KLE cells. The possible reason is that KLE cell line with distinct mutations in TP53 is a model for more aggressive endometrial cancer (Bi et al. 2021; Devor et al. 2020). Considering the possible differential effects of Celastrol on different cell lines, it is worthwhile to explore the role of Celastrol on different cell lines, which requires further study. CDK2, cyclin E and p21 are key proteins that modulates cell cycle progression. CDK2 interacts with cyclin E to induce the G1/S transition, while p21 inhibits cyclin E-CDK2 activity (Harris and Levine 2005). The inhibitory effects of Celastrol on cell viability and cell cycle entry indicated that Celastrol prevented UCEC cell proliferation in vitro. Ki-67 is considered as a proliferative marker in various tumor tissues (Menon et al. 2019). Our findings demonstrated that Celastrol inhibited tumor growth in vivo. In addition, induction of cell apoptosis is considered as a vital way to inhibit tumor growth (Fonseca et al. 2018). TUNEL staining indicated increased cell apoptosis by Celastrol treatment; however, TUNEL-positive cells are not equated with apoptotic cell death. We analyzed the changes in caspase-3 activity, cleaved-caspase-9 and cleaved PARP1 expression in UCEC cells. Caspase-9 is an important protein during the initiation of cell apoptosis, and its activation triggers active caspase-3, and then induces the cleavage of PARP1, an indicator of cells undergoing apoptosis (Green 2022). The increased caspase-3 activity, cleaved-caspase-9 and cleaved PARP1 expression in UCEC cells by treatment of Celastrol further confirmed that Celastrol facilitated apoptosis of UCEC cells. DNA damage and cell apoptosis have a reciprocal relationship. Several investigations demonstrated that unrepaired DNA damage results in cell death via apoptosis (Zio et al. 2013). The comet assay showed obvious DNA damage induced by Celastrol. The result of γ-H2AX, a hallmark of DNA damage, indicated cellular damage caused by Celastrol. The present investigation showed that Celastrol promoted cell apoptosis after DNA damage in UCEC.

Previous study has reported that although Celastrol has remarkable medicinal effects, the side effects limits its clinical application, and identifying the targets of Celastrol action is of great importance (Lim et al. 2021). Through database analysis, we identified KAT2B protein as a possible target of Celastrol. KAT2B was found to inhibit the progression of tumors including esophageal cancer and colorectal cancer (Chen and Allgayer 2023; Cheng et al. 2021). Downregulation of KAT2B expression in UCEC tissues compared to normal tissues suggested that KAT2B might serve an important role in UCEC progression. We found that cell activity and cell cycle entry were promoted in cells with KAT2B knockdown. In contrast, KAT2B overexpression prevented them. The cell apoptosis and DNA damage were enhanced by KAT2B overexpression, which was consistent with the previous study (Chen and Allgayer 2023). After Celastrol treatment, we observed upregulated KAT2B expression in UCEC cells and tumors. The function of Celastrol in promoting DNA damage and apoptosis in UCEC cells was attenuated by knockdown of KAT2B. The report about Celastrol indicated that Celastrol can suppress tumor growth by acting synergistically with histone deacetylase inhibitors (Chen et al. 2022). We concluded that KAT2B might function synergistically with Celastrol to regulate DNA damage and apoptosis of UCEC cells. Further research is needed to investigate whether Celastrol has synergistic effect with KAT2B in UCSC. Moreover, given the known limitations of Celastrol, further exploration of its effective therapeutic approaches is needed in the future, including combination therapy, structural derivatives and nano/micro-systems development. In addition, previous studies have reported that PRDX1, Nur77 and CIP2A are the target proteins of Celastrol (Wu et al. 2023; Xu et al. 2023b; Zhang et al. 2022). It is worth noting that KAT2B is just one of multiple Celastrol-interacting proteins, and our study is the first to reveal that KAT2B is a target of Celastrol. Further investigations are required to clarify the mechanism of Celastrol’s action in UCEC.

Knowing that, we further investigated the molecular mechanism on how KAT2B affects in UCEC cell behaviors. As a lysine acetyltransferase, KAT2B acetylates non-histone target, including transcription factor, to enhance the transcriptional activity of targets (Song et al. 2002). KAT2B’s effect is associated with transcription factor via protein-protein interaction (Ghosh et al. 2018; Song et al. 2002). In UCEC, RBPJ exerts a tumor suppressor role by inhibiting cell malignant behaviors (Xiao et al. 2019). This study demonstrated that KAT2B could bind to the transcription factor RBPJ and mediate its acetylation in UCEC cells, which was consistent with previous finding. Transcription factor RBPJ can act a repressive role through binding of target’s promoter in cells (Xu et al. 2017). There was a negative correlation between RBPJ and MCM4 expression in UCEC tumors by GEPIA analysis. The binding of transcription factor RBPJ and MCM4 promoter was confirmed in our study, and RBPJ overexpression decreased the MCM4 promoter luciferase activity. Previous study has showed that MCM4 functions as a cancer-promoting gene in UCEC (Pei et al. 2022). Our findings showed that MCM4 overexpression induced proliferation, inhibited DNA damage and apoptosis in UCEC cells overexpressing KAT2B. The above results indicated that KAT2B enhanced the transcriptional inhibition ability of RBPJ on MCM4 via promoting RBPJ acetylation. Further analysis involved in Celastrol, RBPJ and MCM4 further indicated that Celastrol functioned in UCEC via KAT2B/RBPJ/MCM4 axis. These results were in line with the previous report that the anti-cancer activity of Celastrol is mediated by downregulating MCM4 expression (Youns and Askoura 2021).

## Conclusion

In conclusion, we identified a potential anti-tumor agent in UCEC, Celastrol. Mechanistically, we uncovered that KAT2B appeared to be a possible target of Celastrol, and Celastrol could upregulate KAT2B to increase the acetylation of RBPJ, promoting the transcriptional inhibition of MCM4, thereby inhibiting cell proliferation, facilitating DNA damage and cell apoptosis. KAT2B might be an important potential molecular target for the treatment of UCEC.

## Electronic supplementary material

Below is the link to the electronic supplementary material.

Fig. S1. KAT2B, RBPJ and MCM4 expression in tumors of UCEC patients. (A–C) The protein expression of KAT2B, RBPJ and MCM4 in UCEC samples from UALCAN database.

Fig. S2. Quantification of γ-H2AX foci. (A–C) The γ-H2AX-positive cells were quantified. All the values are mean ± SD. ∗∗P < 0.01 vs. the control group. ##P < 0.01 vs. the vector group or the Celastrol + shNC group or the KAT2B + vector group.

## Data Availability

No datasets were generated or analysed during the current study.
