# Supplementary figures and images for: Celastrol promotes DNA damage and apoptosis in uterine corpus endometrial carcinoma via promotion of KAT2B-mediated RBPJ acetylation and repression of MCM4 transcription
Source: Mol Med. 2025 Feb 3;31:39. doi: 10.1186/s10020-025-01082-z (PMC11792231; doi:10.1186/s10020-025-01082-z)

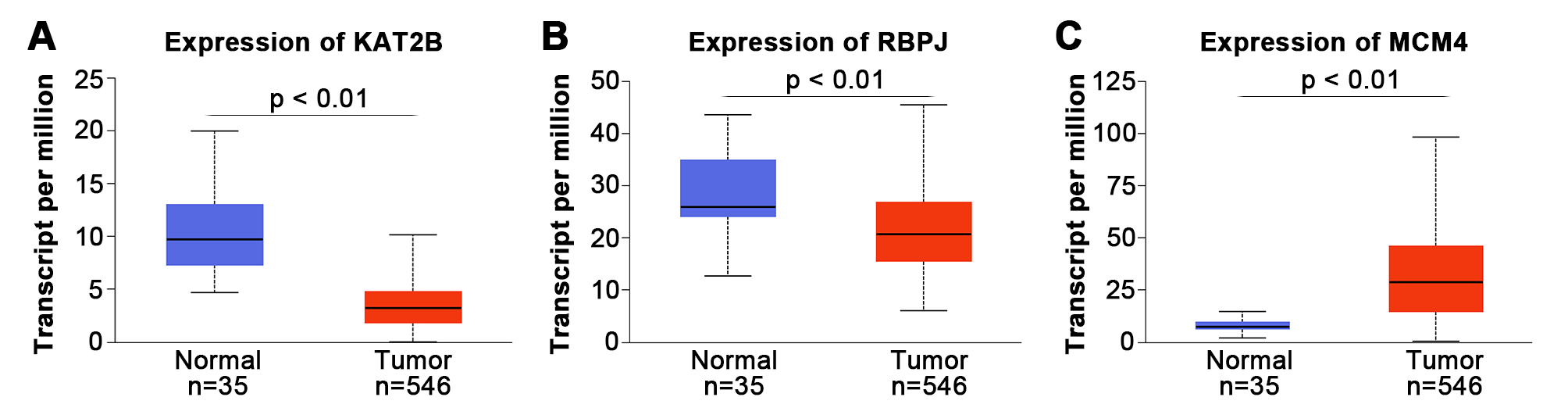

Supplement: Supplementary file 1 — Fig. S1. KAT2B, RBPJ and MCM4 expression in tumors of UCEC patients. (A–C) The protein expression of KAT2B, RBPJ and MCM4 in UCEC samples from UALCAN database. [file 10020_2025_1082_MOESM1_ESM.png]

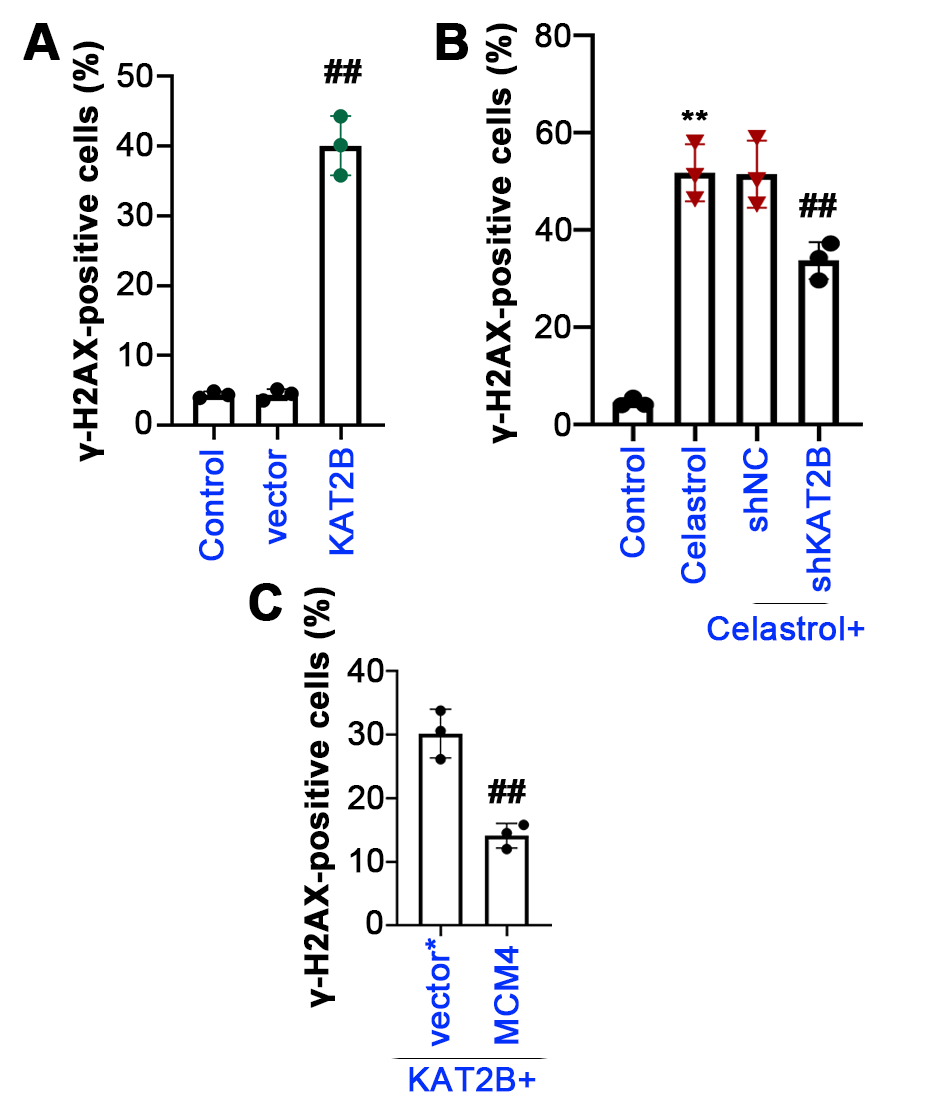

Supplement: Supplementary file 2 — Fig. S2. Quantification of γ-H2AX foci. (A–C) The γ-H2AX-positive cells were quantified. All the values are mean ± SD. ∗∗P < 0.01 vs. the control group. ##P < 0.01 vs. the vector group or the Celastrol + shNC group or the KAT2B + vector group. [file 10020_2025_1082_MOESM2_ESM.png]
